# Supplementary material for: Dysbiosis Triggers ACF Development in Genetically Predisposed Subjects
Source: Cancers (Basel). 2021 Jan 14;13(2):283. doi: 10.3390/cancers13020283 (PMC7828790; doi:10.3390/cancers13020283)
Supplement: Supplementary file 1 [file cancers-13-00283-s001.zip › cancers-1044433-supplemental table.docx]

**Supplementary Material**

Dysbiosis Triggers ACF Development in Genetically Predisposed Subjects

Stefania De Santis, Marina Liso, Mirco Vacca, Giulio Verna, Elisabetta Cavalcanti, Sergio Coletta, Francesco Maria Calabrese, Rajaraman Eri, Antonio Lippolis, Raffaele Armentano, Mauro Mastronardi, Maria De Angelis and Marcello Chieppa

**Table S1.** α-diversity indices.

**A**

| **Genotype** | **Shannon index**  **IQR (median)** | **OTU**  **IQR (median)** |
| --- | --- | --- |
| C57BL/6J | 2.7–2.8 (2.7) | 266.3–305.8 (285) |
| Winnie | 2.8–2.9 (2.9) | 335.7–478.5 (452) |
| APC^Min/+^ | 2.6–2.7 (2.7) | 194–233.3 (213.5) |
| Winnie-APC^Min/+^ | 2.5–2.7 (2.7) | 289–346.5 (314) |

**B**

|  | **Shannon index**  ***p*** | **OTU**  ***p*** |
| --- | --- | --- |
| C57BL/6J vs. Winnie | NS* | NS* |
| C57BL/6J vs. APC^Min/+^ | NS* | NS* |
| C57BL/6J vs. Winnie-APC^Min/+^ | NS* | NS* |
| Winnie vs. APC^Min/+^ | 0.0286 | NS* |
| APC^Min/+^ vs. Winnie-APC^Min/+^ | NS* | NS* |
| Winnie vs. Winnie-APC^Min/+^ | 0.0242 | 0.0167 |

*NS: not significant

(**A**) α-diversity was evaluated as Shannon index and OTU number in fecal samples of 8-week-old C57BL/6J (wild type, WT), Winnie, APC^Min/+^ and Winnie-APC^Min/+^ mice. (**B**) Significant differences (p values < 0.05; Mann-Whitney test) between different sampled groups were reported as median and inter-quartile range (IQR; 25^th^ - 75^th^ percentile).
