# Supplementary material for: Dysbiosis Triggers ACF Development in Genetically Predisposed Subjects
Source: Cancers (Basel). 2021 Jan 14;13(2):283. doi: 10.3390/cancers13020283 (PMC7828790; doi:10.3390/cancers13020283)
Supplement: Supplementary file 1 [file cancers-13-00283-s001.zip › supplemental figures/Figure S2.pdf]

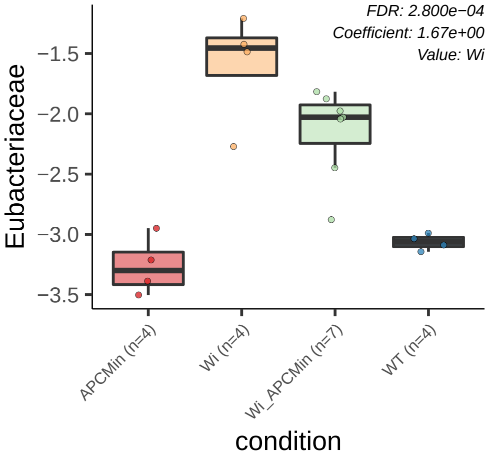

FDR: 2.800e−04
Coefficient: 1.67e+00
Value: Wi
Eubacteriaceae
−1.5
−2.0
−2.5
−3.0
−3.5
APCMin (n=4)
Wi (n=4)
Wi_APCMin (n=7)
WT (n=4)
condition

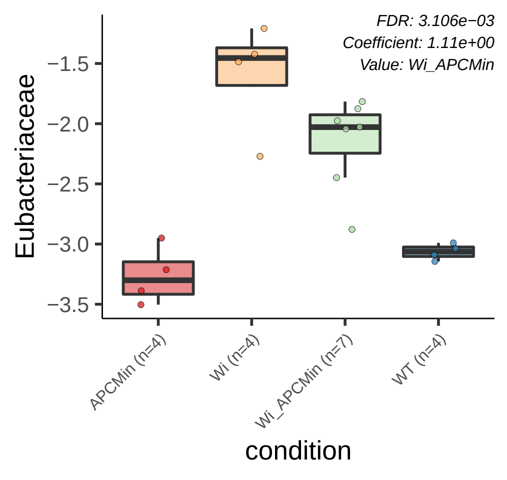
FDR: 3.106e−03
Coefficient: 1.11e+00
Value: Wi_APCMin
Eubacteriaceae
−1.5
−2.0
−2.5
−3.0
−3.5
APCMin (n=4)
Wi (n=4)
Wi_APCMin (n=7)
WT (n=4)
condition

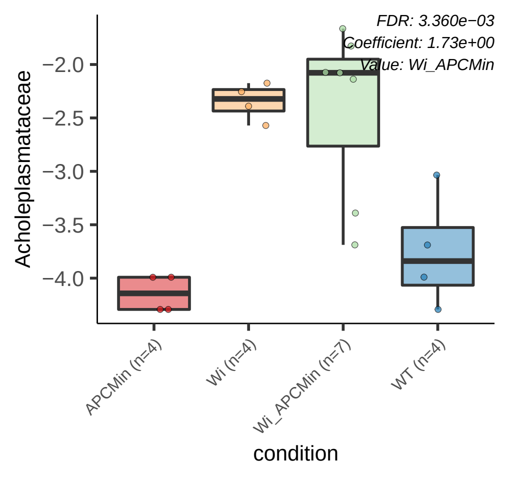

FDR: 3.360e−03
Coefficient: 1.73e+00
Value: Wi_APCMin
Acholeplasmataceae
−2.0
−2.5
−3.0
−3.5
−4.0
APCMin (n=4)
Wi (n=4)
Wi_APCMin (n=7)
WT (n=4)
condition

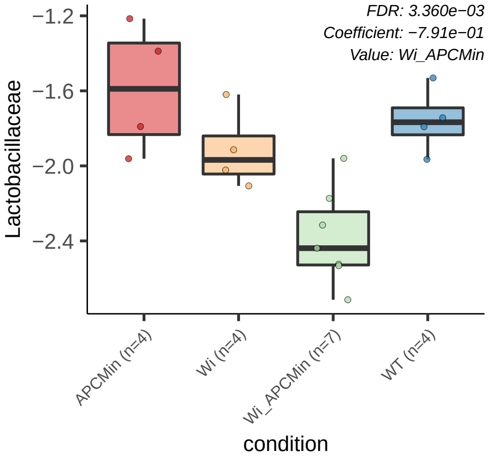

FDR: 3.360e−03
Coefficient: −7.91e−01
Value: Wi_APCMin
Lactobacillaceae
−1.2
−1.6
−2.0
−2.4
APCMin (n=4)
Wi (n=4)
Wi_APCMin (n=7)
WT (n=4)
condition

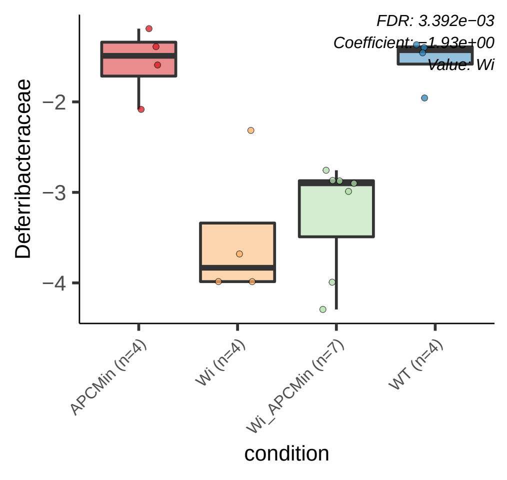
FDR: 3.392e−03
Coefficient: −1.93e+00
Value: Wi
Deferribacteraceae
−2
−3
−4
APCMin (n=4)
Wi (n=4)
Wi_APCMin (n=7)
WT (n=4)
condition

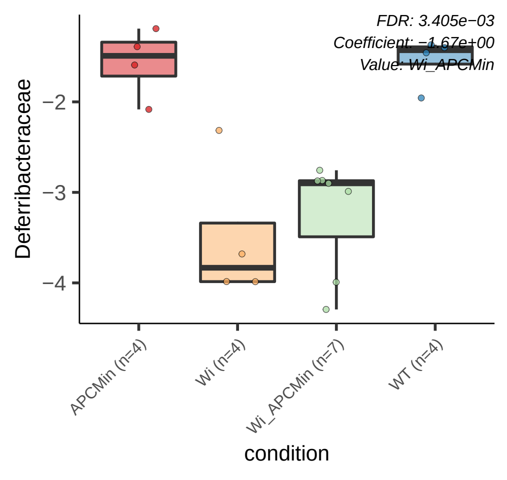
FDR: 3.405e−03
Coefficient: −1.67e+00
Value: Wi_APCMin
Deferribacteraceae
−2
−3
−4
APCMin (n=4)
Wi (n=4)
Wi_APCMin (n=7)
WT (n=4)
condition

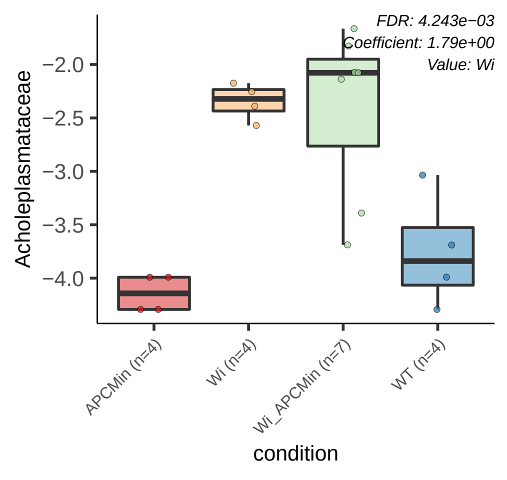

FDR: 4.243e−03
Coefficient: 1.79e+00
Value: Wi
Acholeplasmataceae
−2.0
−2.5
−3.0
−3.5
−4.0
APCMin (n=4)
Wi (n=4)
Wi_APCMin (n=7)
WT (n=4)
condition

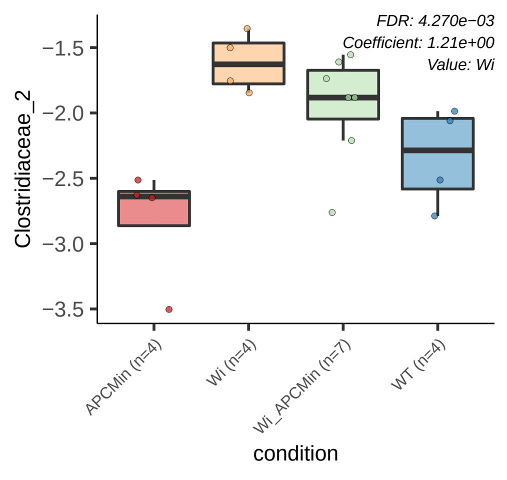

FDR: 4.270e−03
Coefficient: 1.21e+00
Value: Wi
Clostridiaceae_2
−1.5
−2.0
−2.5
−3.0
−3.5
APCMin (n=4)
Wi (n=4)
Wi_APCMin (n=7)
WT (n=4)
condition

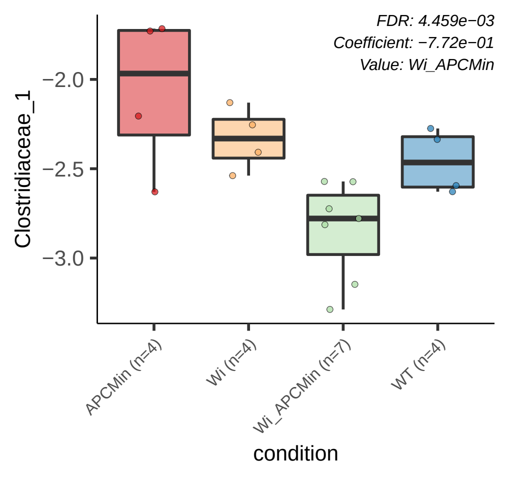

FDR: 4.459e−03
Coefficient: −7.72e−01
Value: Wi_APCMin
Clostridiaceae_1
−2.0
−2.5
−3.0
APCMin (n=4)
Wi (n=4)
Wi_APCMin (n=7)
WT (n=4)
condition

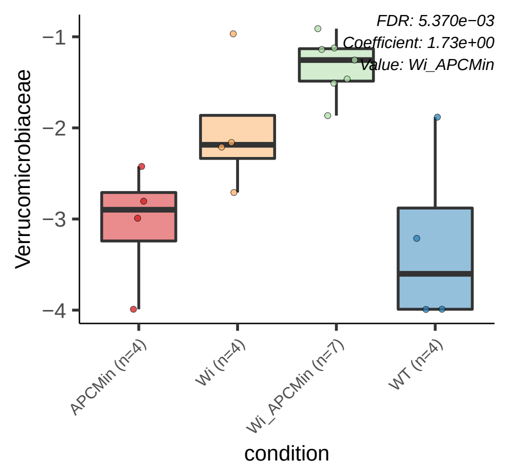
FDR: 5.370e−03
Coefficient: 1.73e+00
Value: Wi_APCMin
Verrucomicrobiaceae
−1
−2
−3
−4
APCMin (n=4)
Wi (n=4)
Wi_APCMin (n=7)
WT (n=4)
condition

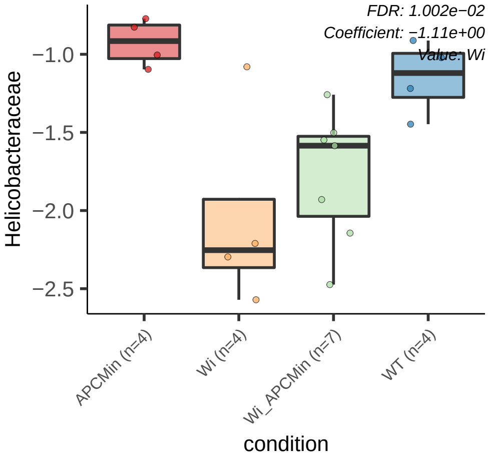
FDR: 1.002e−02
Coefficient: −1.11e+00
Value: Wi
Helicobacteraceae
−1.0
−1.5
−2.0
−2.5
APCMin (n=4)
Wi (n=4)
Wi_APCMin (n=7)
WT (n=4)
condition

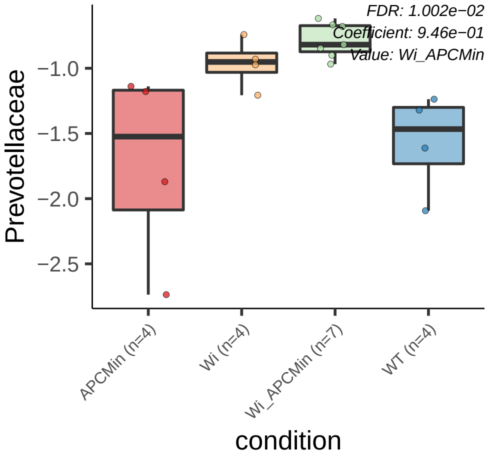
FDR: 1.002e−02
Coefficient: 9.46e−01
Value: Wi_APCMin
Prevotellaceae
−1.0
−1.5
−2.0
−2.5
APCMin (n=4)
Wi (n=4)
Wi_APCMin (n=7)
WT (n=4)
condition

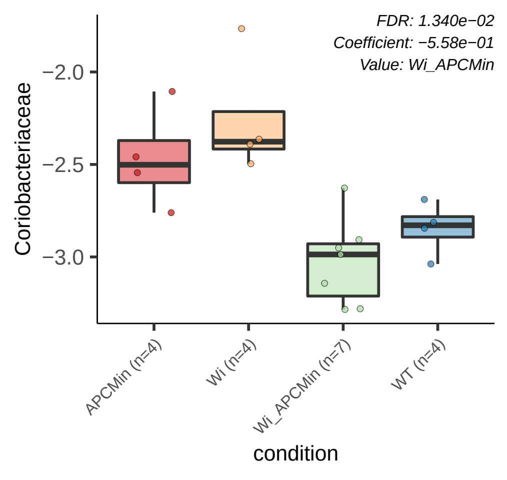
FDR: 1.340e−02
Coefficient: −5.58e−01
Value: Wi_APCMin
−2.0
−2.5
−3.0
Coriobacteriaceae
APCMin (n=4)
Wi (n=4)
Wi_APCMin (n=7)
WT (n=4)
condition

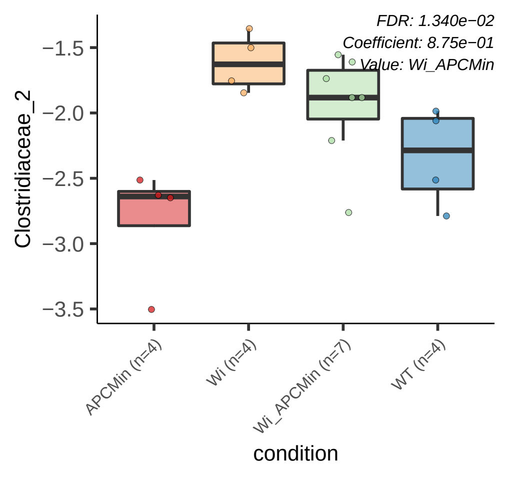
FDR: 1.340e−02
Coefficient: 8.75e−01
Value: Wi_APCMin
Clostridiaceae_2
−1.5
−2.0
−2.5
−3.0
−3.5
APCMin (n=4)
Wi (n=4)
Wi_APCMin (n=7)
WT (n=4)
condition

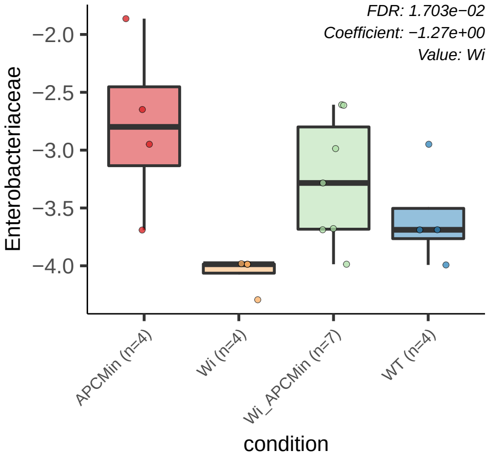
FDR: 1.703e−02
Coefficient: −1.27e+00
Value: Wi
Enterobacteriaceae
−2.0
−2.5
−3.0
−3.5
−4.0
APCMin (n=4)
Wi (n=4)
Wi_APCMin (n=7)
WT (n=4)
condition

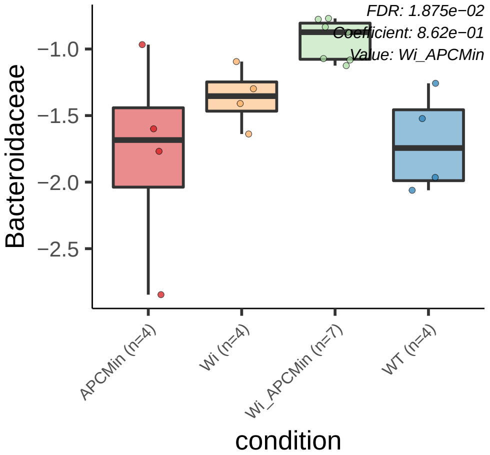

FDR: 1.875e−02
Coefficient: 8.62e−01
Value: Wi_APCMin
Bacteroidaceae
−1.0
−1.5
−2.0
−2.5
APCMin (n=4)
Wi (n=4)
Wi_APCMin (n=7)
WT (n=4)
condition

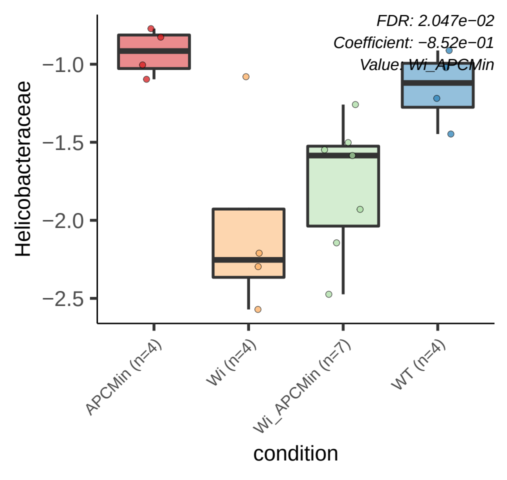
FDR: 2.047e−02
Coefficient: −8.52e−01
Value: Wi_APCMin
Helicobacteraceae
−1.0
−1.5
−2.0
−2.5
APCMin (n=4)
Wi (n=4)
Wi_APCMin (n=7)
WT (n=4)
condition

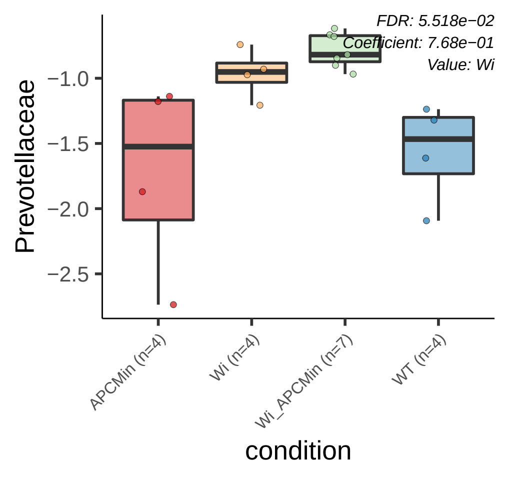

FDR: 5.518e−02
Coefficient: 7.68e−01
Value: Wi
Prevotellaceae
−1.0
−1.5
−2.0
−2.5
APCMin (n=4)
Wi (n=4)
Wi_APCMin (n=7)
WT (n=4)
condition

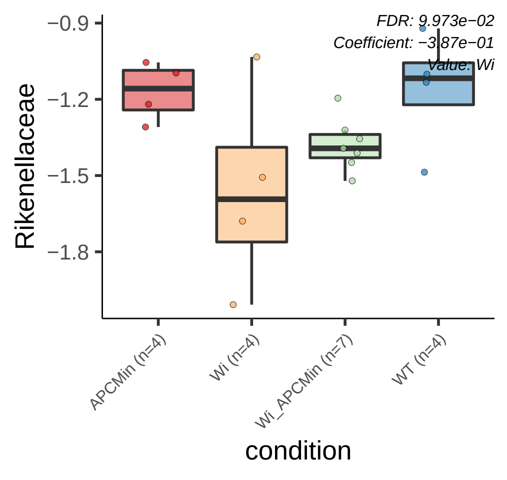

FDR: 9.973e−02
Coefficient: −3.87e−01
Value: Wi
Rikenellaceae
−0.9
−1.2
−1.5
−1.8
APCMin (n=4)
Wi (n=4)
Wi_APCMin (n=7)
WT (n=4)
condition

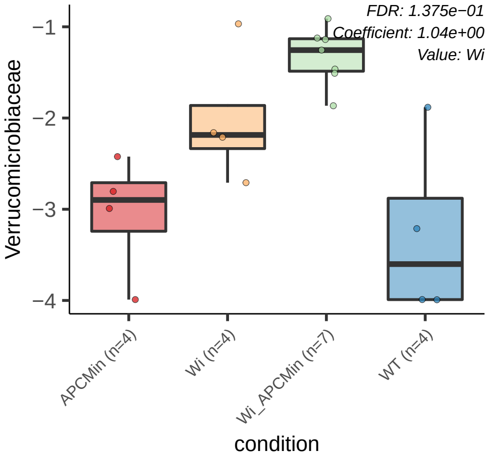

FDR: 1.375e−01
Coefficient: 1.04e+00
Value: Wi
Verrucomicrobiaceae
−1
−2
−3
−4
APCMin (n=4)
Wi (n=4)
Wi_APCMin (n=7)
WT (n=4)
condition

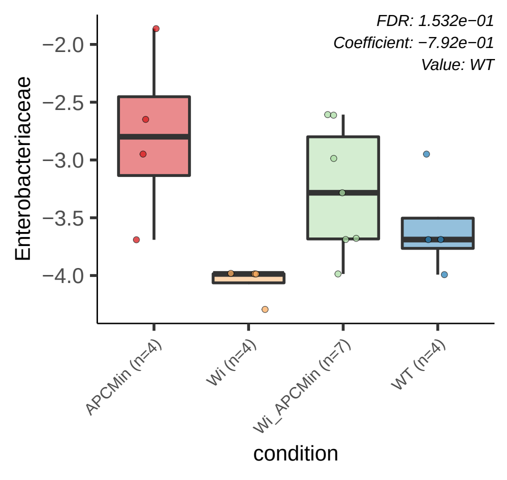

FDR: 1.532e−01
Coefficient: −7.92e−01
Value: WT
Enterobacteriaceae
−2.0
−2.5
−3.0
−3.5
−4.0
APCMin (n=4)
Wi (n=4)
Wi_APCMin (n=7)
WT (n=4)
condition

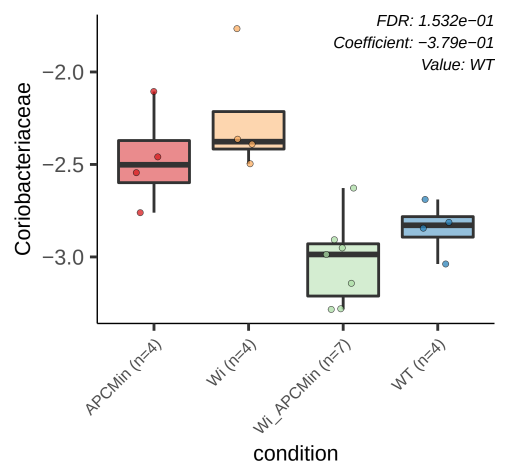

FDR: 1.532e−01
Coefficient: −3.79e−01
Value: WT
Coriobacteriaceae
−2.0
−2.5
−3.0
APCMin (n=4)
Wi (n=4)
Wi_APCMin (n=7)
WT (n=4)
condition

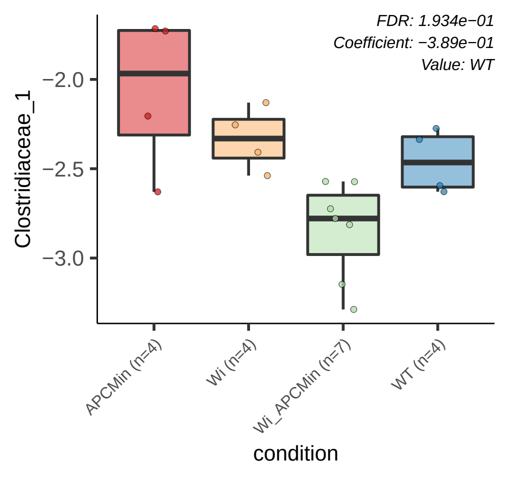
FDR: 1.934e−01
Coefficient: −3.89e−01
Value: WT
Clostridiaceae_1
−2.0
−2.5
−3.0
APCMin (n=4)
Wi (n=4)
Wi_APCMin (n=7)
WT (n=4)
condition

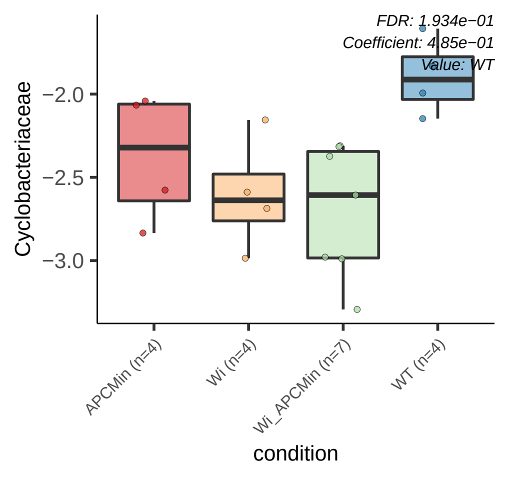

FDR: 1.934e−01
Coefficient: 4.85e−01
Value: WT
Cyclobacteriaceae
−2.0
−2.5
−3.0
APCMin (n=4)
Wi (n=4)
Wi_APCMin (n=7)
WT (n=4)
condition

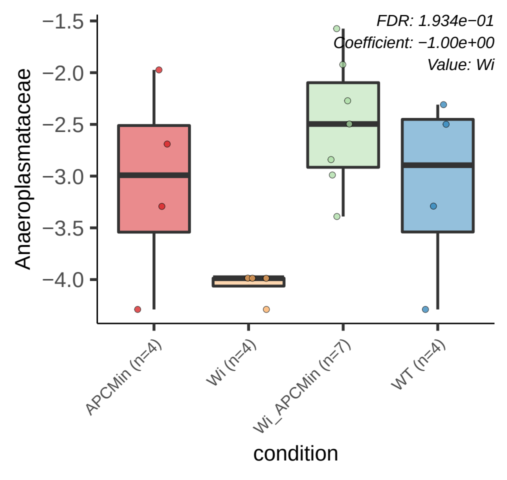

FDR: 1.934e−01
Coefficient: −1.00e+00
Value: Wi
Anaeroplasmataceae
−1.5
−2.0
−2.5
−3.0
−3.5
−4.0
APCMin (n=4)
Wi (n=4)
Wi_APCMin (n=7)
WT (n=4)
condition

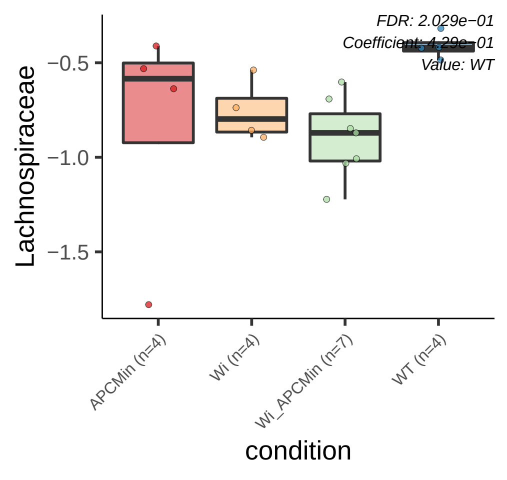

FDR: 2.029e−01
Coefficient: 4.29e−01
Value: WT
Lachnospiraceae
−0.5
−1.0
−1.5
APCMin (n=4)
Wi (n=4)
Wi_APCMin (n=7)
WT (n=4)
condition

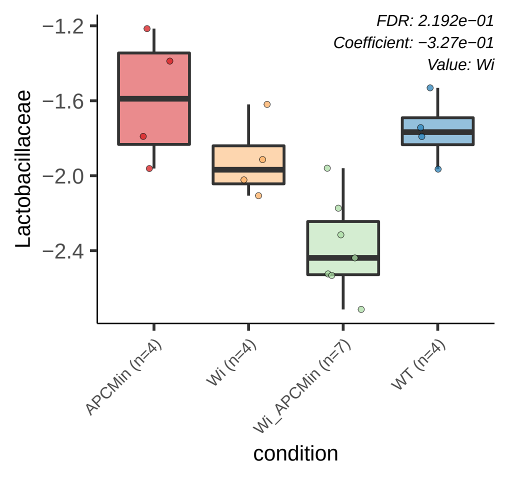

FDR: 2.192e−01
Coefficient: −3.27e−01
Value: Wi
Lactobacillaceae
−1.2
−1.6
−2.0
−2.4
APCMin (n=4)
Wi (n=4)
Wi_APCMin (n=7)
WT (n=4)
condition

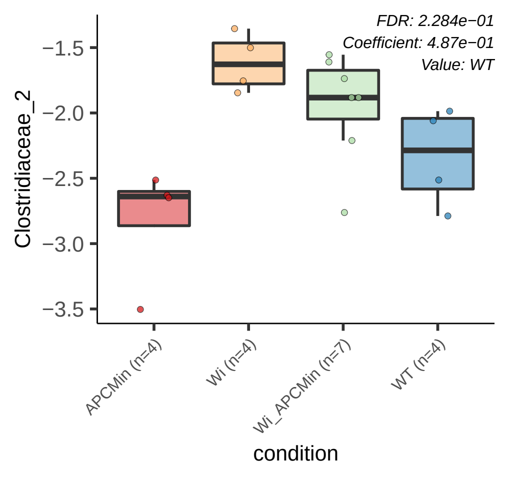

FDR: 2.284e−01
Coefficient: 4.87e−01
Value: WT
Clostridiaceae_2
−1.5
−2.0
−2.5
−3.0
−3.5
APCMin (n=4)
Wi (n=4)
Wi_APCMin (n=7)
WT (n=4)
condition

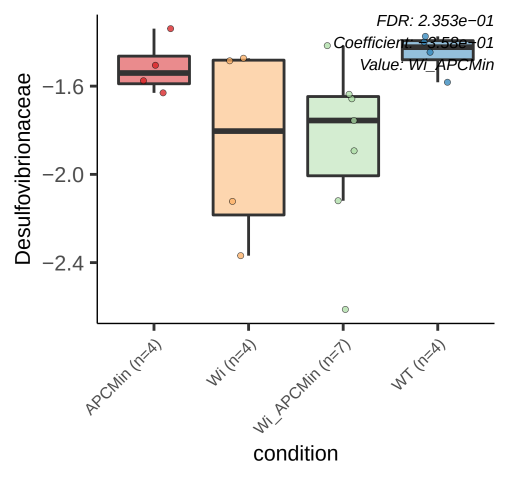
FDR: 2.353e−01
Coefficient: −3.58e−01
Value: Wi_APCMin
Desulfovibrionaceae
−1.6
−2.0
−2.4
APCMin (n=4)
Wi (n=4)
Wi_APCMin (n=7)
WT (n=4)
condition

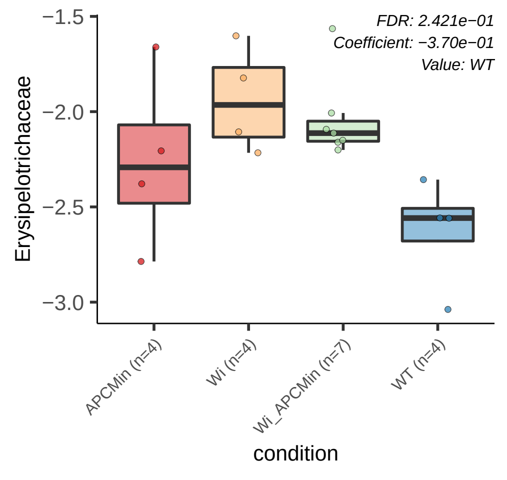
FDR: 2.421e−01
Coefficient: −3.70e−01
Value: WT
Erysipelotrichaceae
−1.5
−2.0
−2.5
−3.0
APCMin (n=4)
Wi (n=4)
Wi_APCMin (n=7)
WT (n=4)
condition

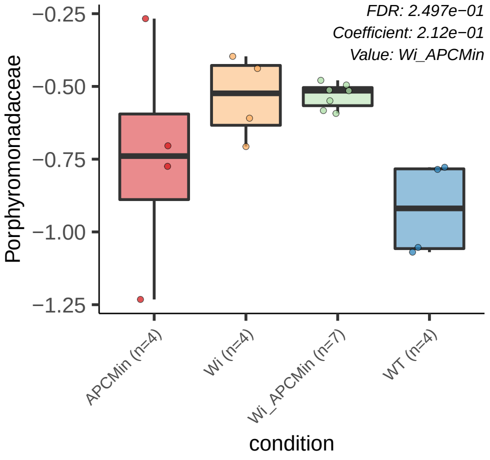

FDR: 2.497e−01
Coefficient: 2.12e−01
Value: Wi_APCMin
Porphyromonadaceae
−0.25
−0.50
−0.75
−1.00
−1.25
APCMin (n=4)
Wi (n=4)
Wi_APCMin (n=7)
WT (n=4)
condition
