# Supplementary material for: Dysbiosis Triggers ACF Development in Genetically Predisposed Subjects
Source: Cancers (Basel). 2021 Jan 14;13(2):283. doi: 10.3390/cancers13020283 (PMC7828790; doi:10.3390/cancers13020283)
Supplement: Supplementary file 1 [file cancers-13-00283-s001.zip › supplemental figures/Figure S3.pdf]

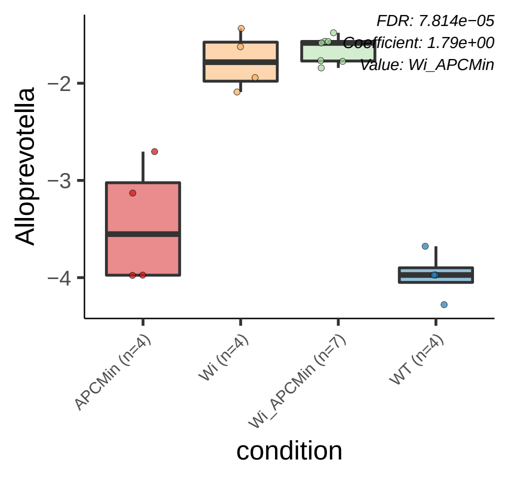

FDR: 7.814e−05
Coefficient: 1.79e+00
Value: Wi_APCMin
Alloprevotella
−2
−3
−4
APCMin (n=4)
Wi (n=4)
Wi_APCMin (n=7)
WT (n=4)
condition

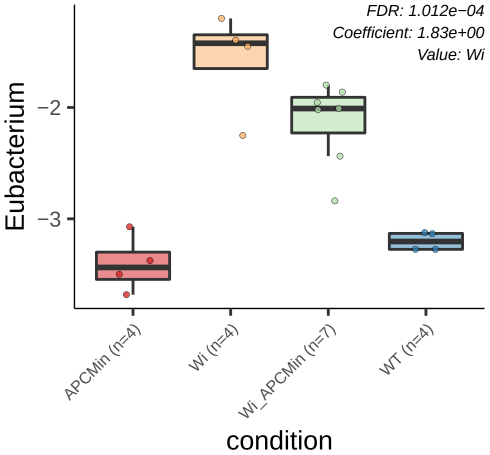

FDR: 1.012e−04
Coefficient: 1.83e+00
Value: Wi
Eubacterium
−2
−3
APCMin (n=4)
Wi (n=4)
Wi_APCMin (n=7)
WT (n=4)
condition

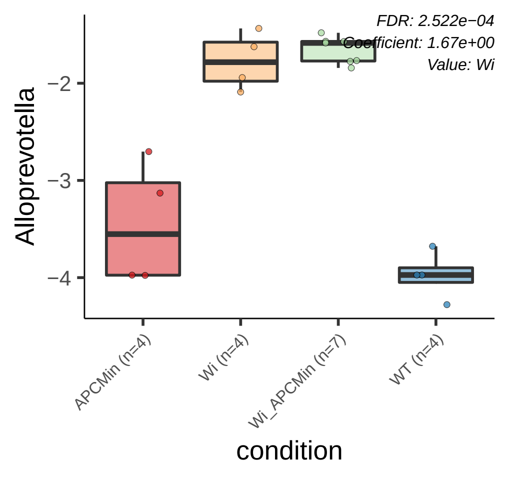

FDR: 2.522e−04
Coefficient: 1.67e+00
Value: Wi
Alloprevotella
−2
−3
−4
APCMin (n=4)
Wi (n=4)
Wi_APCMin (n=7)
WT (n=4)
condition

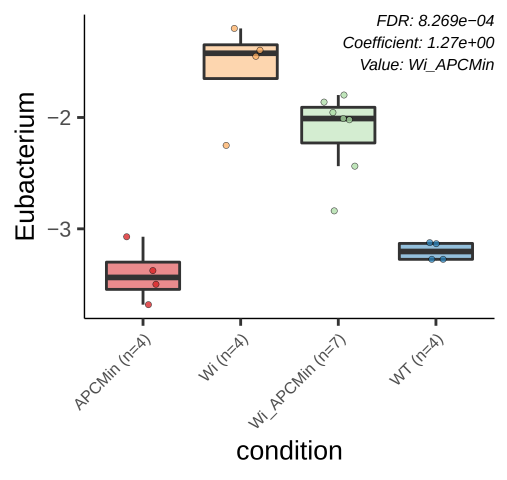

FDR: 8.269e−04
Coefficient: 1.27e+00
Value: Wi_APCMin
Eubacterium
−2
−3
APCMin (n=4)
Wi (n=4)
Wi_APCMin (n=7)
WT (n=4)
condition

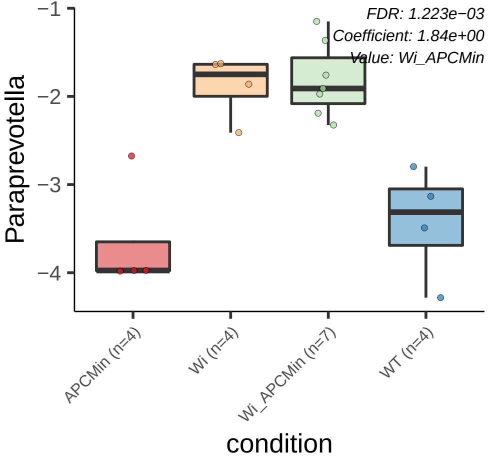

FDR: 1.223e−03
Coefficient: 1.84e+00
Value: Wi_APCMin
Paraprevotella
−1
−2
−3
−4
APCMin (n=4)
Wi (n=4)
Wi_APCMin (n=7)
WT (n=4)
condition

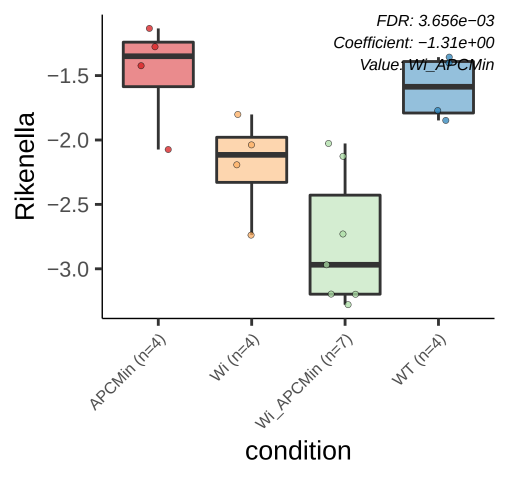
FDR: 3.656e−03
Coefficient: −1.31e+00
Value: Wi_APCMin
Rikenella
−1.5
−2.0
−2.5
−3.0
APCMin (n=4)
Wi (n=4)
Wi_APCMin (n=7)
WT (n=4)
condition

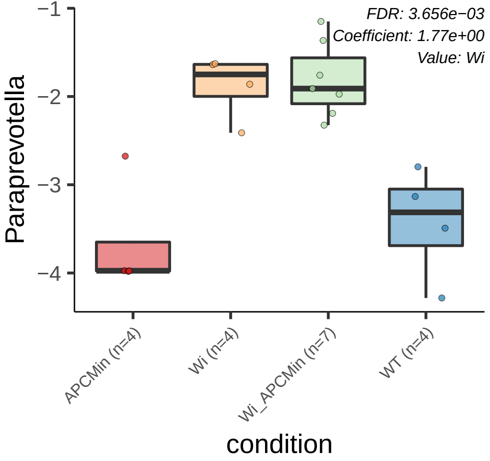
FDR: 3.656e−03
Coefficient: 1.77e+00
Value: Wi
Paraprevotella
−1
−2
−3
−4
APCMin (n=4)
Wi (n=4)
Wi_APCMin (n=7)
WT (n=4)
condition

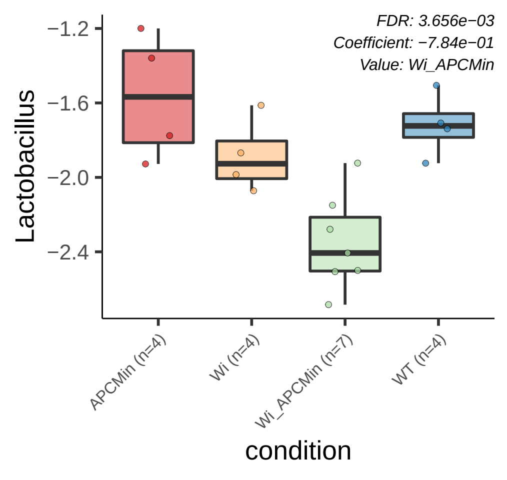
FDR: 3.656e−03
Coefficient: −7.84e−01
Value: Wi_APCMin
Lactobacillus
−1.2
−1.6
−2.0
−2.4
APCMin (n=4)
Wi (n=4)
Wi_APCMin (n=7)
WT (n=4)
condition

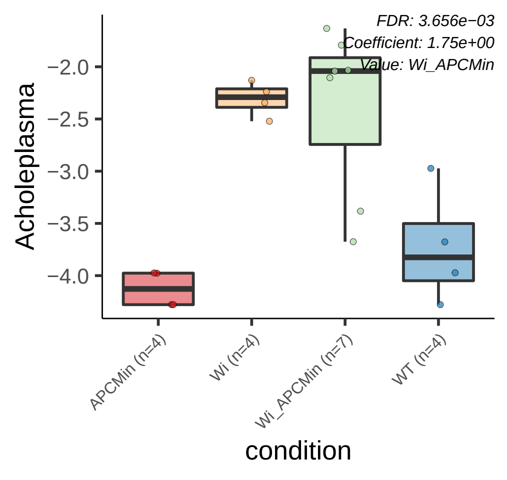

FDR: 3.656e−03
Coefficient: 1.75e+00
Value: Wi_APCMin
Acholeplasma
−2.0
−2.5
−3.0
−3.5
−4.0
APCMin (n=4)
Wi (n=4)
Wi_APCMin (n=7)
WT (n=4)
condition

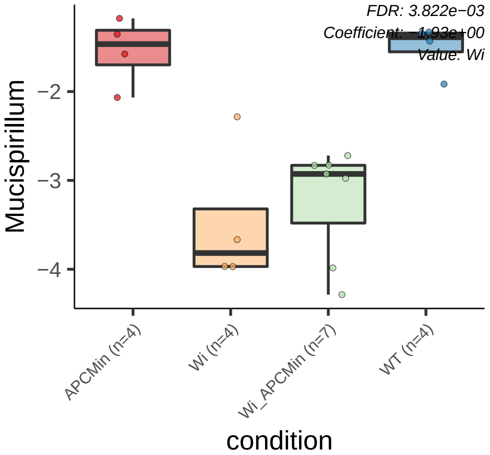
FDR: 3.822e−03
Coefficient: −1.93e+00
Value: Wi
Mucispirillum
−2
−3
−4
APCMin (n=4)
Wi (n=4)
Wi_APCMin (n=7)
WT (n=4)
condition

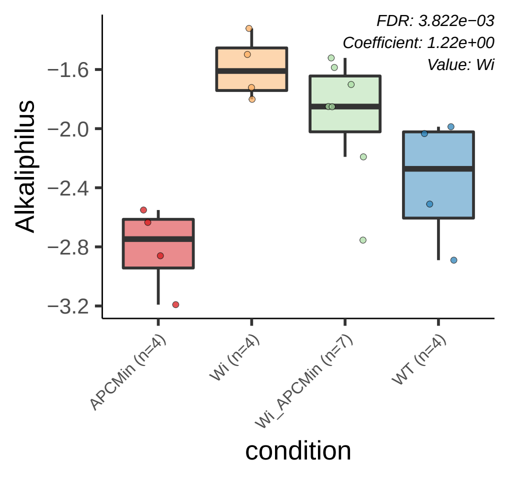
FDR: 3.822e−03
Coefficient: 1.22e+00
Value: Wi
Alkaliphilus
−1.6
−2.0
−2.4
−2.8
−3.2
APCMin (n=4)
Wi (n=4)
Wi_APCMin (n=7)
WT (n=4)
condition

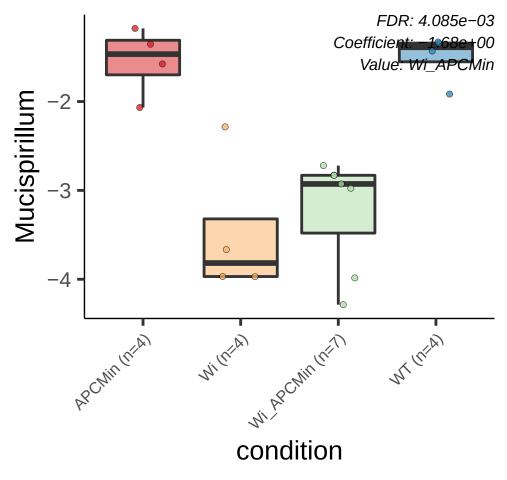
FDR: 4.085e−03
Coefficient: −1.68e+00
Value: Wi_APCMin
Mucispirillum
−2
−3
−4
APCMin (n=4)
Wi (n=4)
Wi_APCMin (n=7)
WT (n=4)
condition

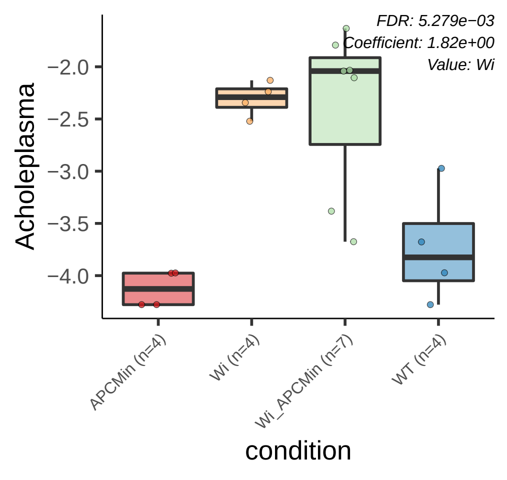

FDR: 5.279e−03
Coefficient: 1.82e+00
Value: Wi
Acholeplasma
−2.0
−2.5
−3.0
−3.5
−4.0
APCMin (n=4)
Wi (n=4)
Wi_APCMin (n=7)
WT (n=4)
condition

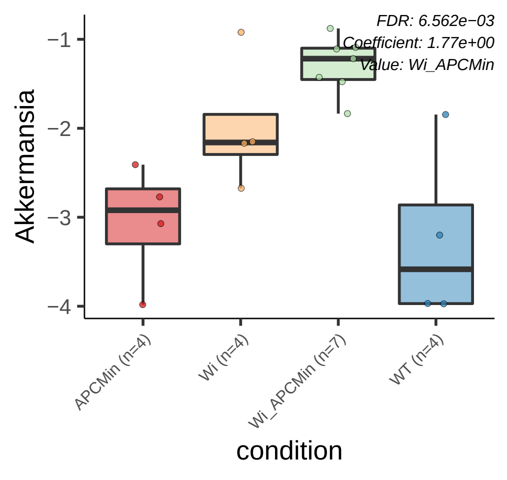
FDR: 6.562e−03
Coefficient: 1.77e+00
Value: Wi_APCMin
Akkermansia
−1
−2
−3
−4
APCMin (n=4)
Wi (n=4)
Wi_APCMin (n=7)
WT (n=4)
condition

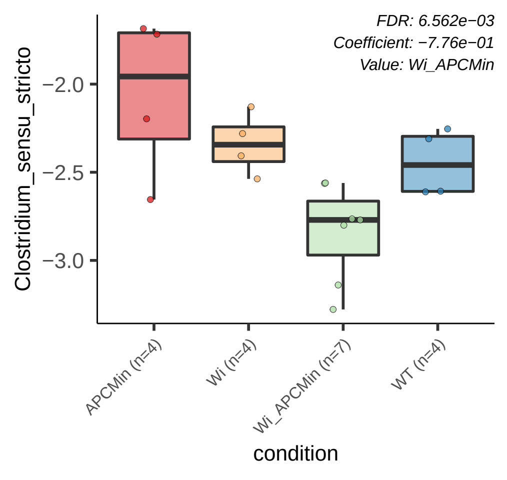

FDR: 6.562e−03
Coefficient: −7.76e−01
Value: Wi_APCMin
Clostridium_sensu_stricto
−2.0
−2.5
−3.0
APCMin (n=4)
Wi (n=4)
Wi_APCMin (n=7)
WT (n=4)
condition

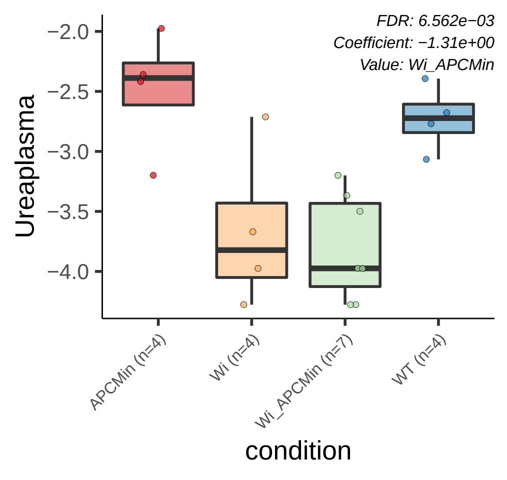
FDR: 6.562e−03
Coefficient: −1.31e+00
Value: Wi_APCMin
Ureaplasma
−2.0
−2.5
−3.0
−3.5
−4.0
APCMin (n=4)
Wi (n=4)
Wi_APCMin (n=7)
WT (n=4)
condition

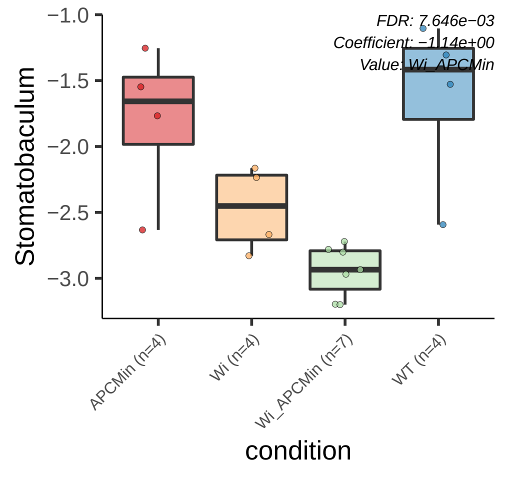

FDR: 7.646e−03
Coefficient: −1.14e+00
Value: Wi_APCMin
Stomatobaculum
−1.0
−1.5
−2.0
−2.5
−3.0
APCMin (n=4)
Wi (n=4)
Wi_APCMin (n=7)
WT (n=4)
condition

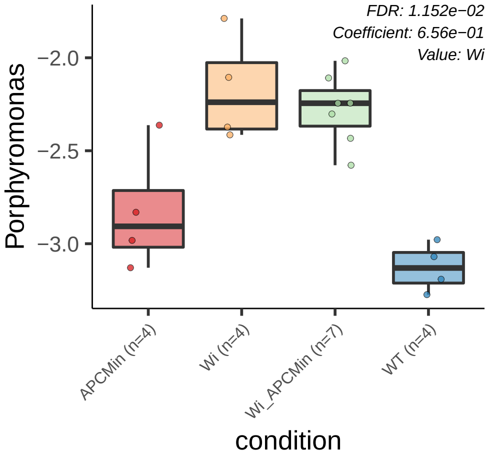

FDR: 1.152e−02
Coefficient: 6.56e−01
Value: Wi
Porphyromonas
−2.0
−2.5
−3.0
APCMin (n=4)
Wi (n=4)
Wi_APCMin (n=7)
WT (n=4)
condition

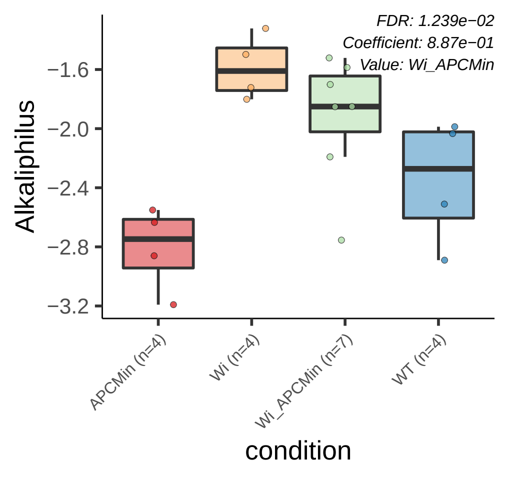

FDR: 1.239e−02
Coefficient: 8.87e−01
Value: Wi_APCMin
Alkaliphilus
−1.6
−2.0
−2.4
−2.8
−3.2
APCMin (n=4)
Wi (n=4)
Wi_APCMin (n=7)
WT (n=4)
condition

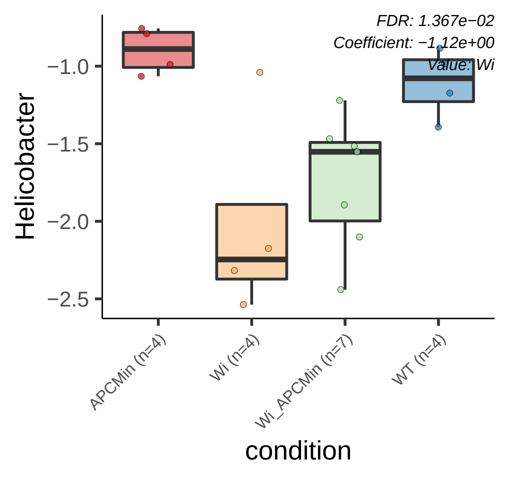

FDR: 1.367e−02
Coefficient: −1.12e+00
Value: Wi
Helicobacter
−1.0
−1.5
−2.0
−2.5
APCMin (n=4)
Wi (n=4)
Wi_APCMin (n=7)
WT (n=4)
condition

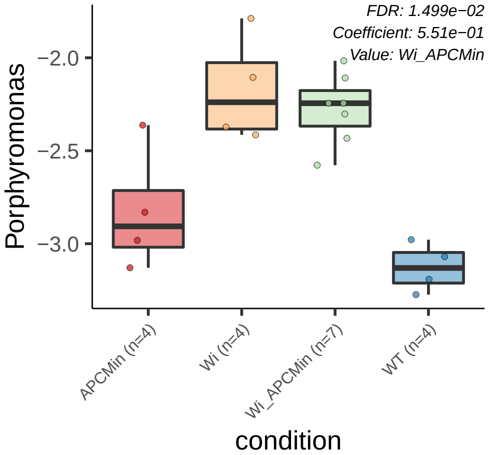

FDR: 1.499e−02
Coefficient: 5.51e−01
Value: Wi_APCMin
Porphyromonas
−2.0
−2.5
−3.0
APCMin (n=4)
Wi (n=4)
Wi_APCMin (n=7)
WT (n=4)
condition

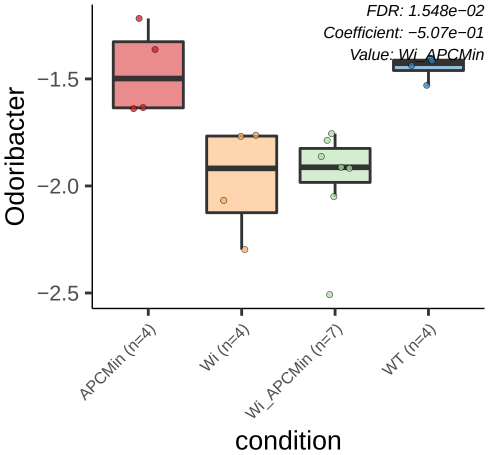

FDR: 1.548e−02
Coefficient: −5.07e−01
Value: Wi_APCMin
Odoribacter
−1.5
−2.0
−2.5
APCMin (n=4)
Wi (n=4)
Wi_APCMin (n=7)
WT (n=4)
condition

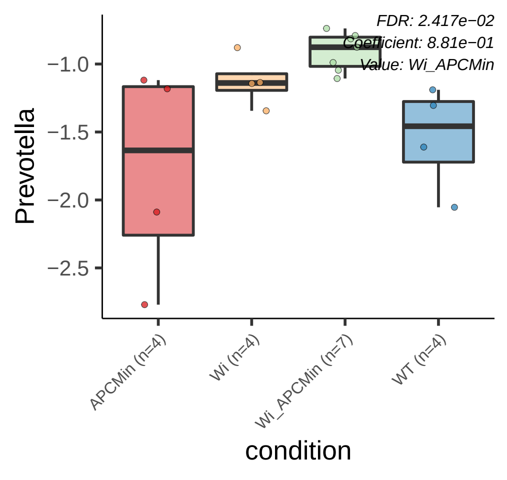

FDR: 2.417e−02
Coefficient: 8.81e−01
Value: Wi_APCMin
Prevotella
−1.0
−1.5
−2.0
−2.5
APCMin (n=4)
Wi (n=4)
Wi_APCMin (n=7)
WT (n=4)
condition

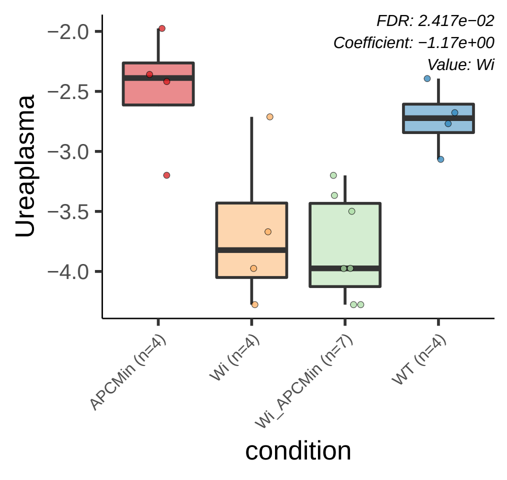

FDR: 2.417e−02
Coefficient: −1.17e+00
Value: Wi
Ureaplasma
−2.0
−2.5
−3.0
−3.5
−4.0
APCMin (n=4)
Wi (n=4)
Wi_APCMin (n=7)
WT (n=4)
condition

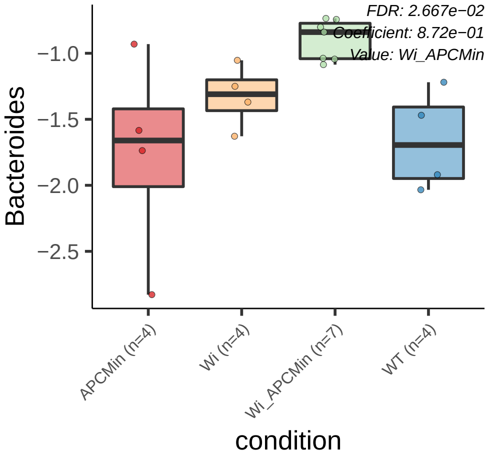
FDR: 2.667e−02
Coefficient: 8.72e−01
Value: Wi_APCMin
Bacteroides
−1.0
−1.5
−2.0
−2.5
APCMin (n=4)
Wi (n=4)
Wi_APCMin (n=7)
WT (n=4)
condition

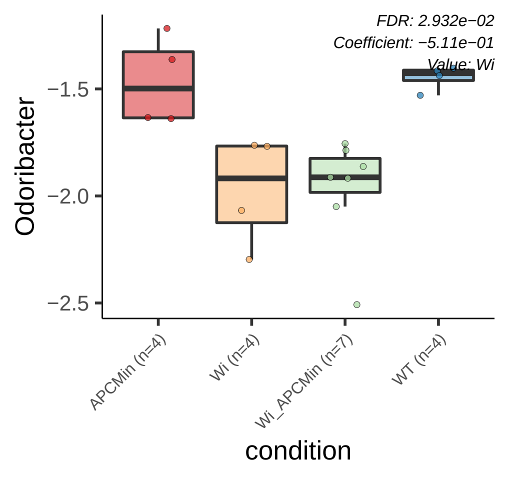
FDR: 2.932e−02
Coefficient: −5.11e−01
Value: Wi
Odoribacter
−1.5
−2.0
−2.5
APCMin (n=4)
Wi (n=4)
Wi_APCMin (n=7)
WT (n=4)
condition

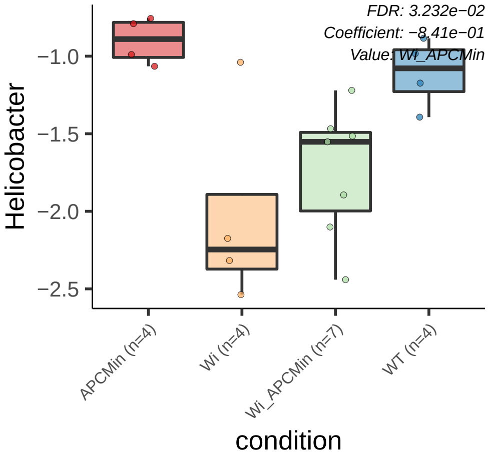

FDR: 3.232e−02
Coefficient: −8.41e−01
Value: Wi_APCMin
Helicobacter
−1.0
−1.5
−2.0
−2.5
APCMin (n=4)
Wi (n=4)
Wi_APCMin (n=7)
WT (n=4)
condition

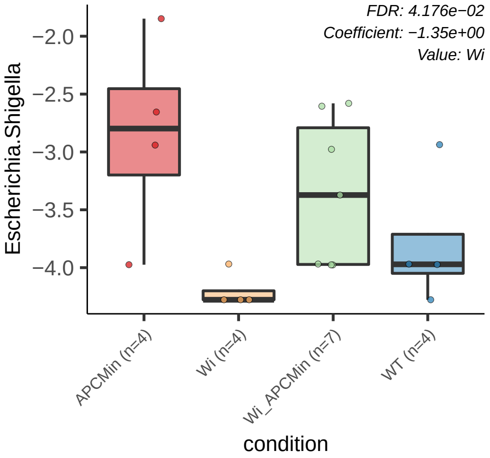

FDR: 4.176e−02
Coefficient: −1.35e+00
Value: Wi
Escherichia.Shigella
−2.0
−2.5
−3.0
−3.5
−4.0
APCMin (n=4)
Wi (n=4)
Wi_APCMin (n=7)
WT (n=4)
condition

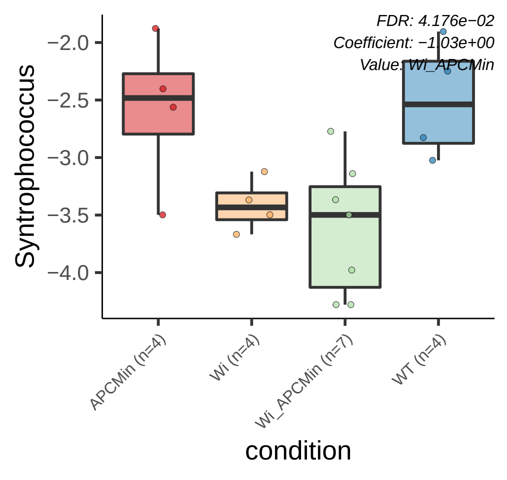

FDR: 4.176e−02
Coefficient: −1.03e+00
Value: Wi_APCMin
Syntrophococcus
−2.0
−2.5
−3.0
−3.5
−4.0
APCMin (n=4)
Wi (n=4)
Wi_APCMin (n=7)
WT (n=4)
condition

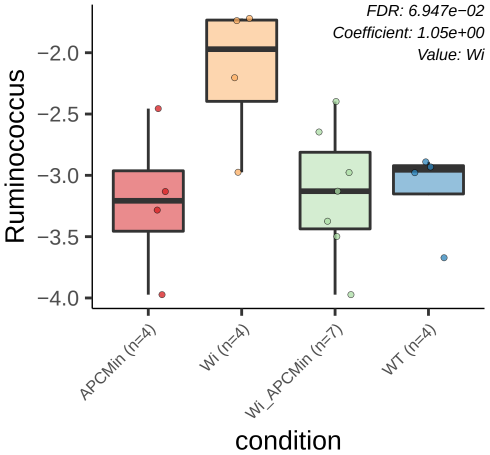

FDR: 6.947e−02
Coefficient: 1.05e+00
Value: Wi
Ruminococcus
−2.0
−2.5
−3.0
−3.5
−4.0
APCMin (n=4)
Wi (n=4)
Wi_APCMin (n=7)
WT (n=4)
condition

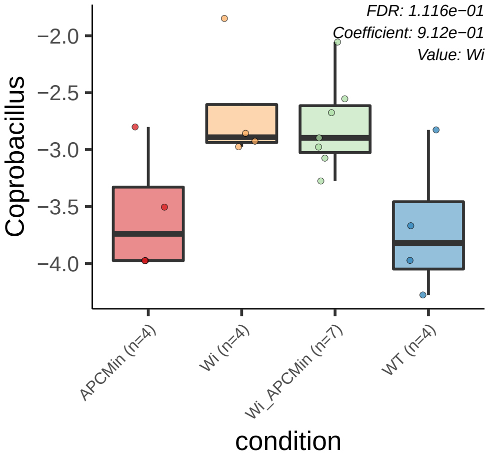

FDR: 1.116e−01
Coefficient: 9.12e−01
Value: Wi
Coprobacillus
−2.0
−2.5
−3.0
−3.5
−4.0
APCMin (n=4)
Wi (n=4)
Wi_APCMin (n=7)
WT (n=4)
condition

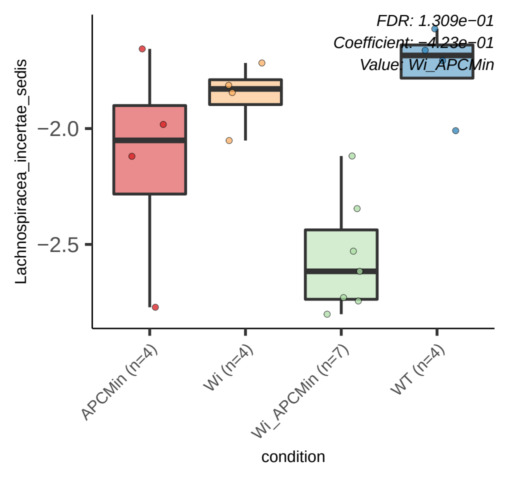

FDR: 1.309e−01
Coefficient: −4.23e−01
Value: Wi_APCMin
Lachnospiracea_incertae_sedis
−2.0
−2.5
APCMin (n=4)
Wi (n=4)
Wi_APCMin (n=7)
WT (n=4)
condition

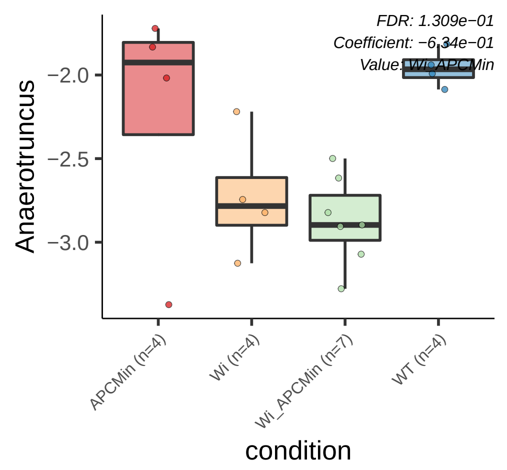
FDR: 1.309e−01
Coefficient: −6.34e−01
Value: Wi_APCMin
Anaerotruncus
−2.0
−2.5
−3.0
APCMin (n=4)
Wi (n=4)
Wi_APCMin (n=7)
WT (n=4)
condition

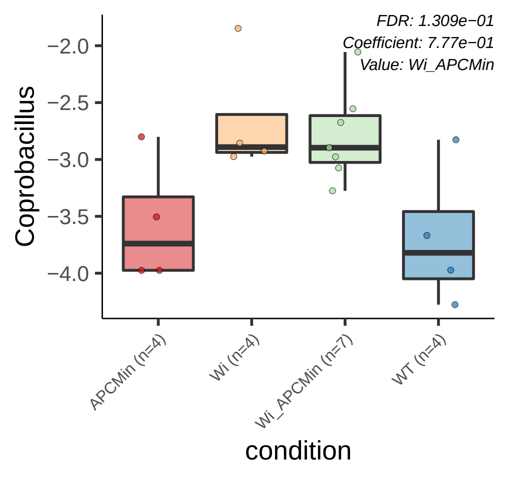

FDR: 1.309e−01
Coefficient: 7.77e−01
Value: Wi_APCMin
−2.0
−2.5
−3.0
−3.5
−4.0
Coprobacillus
APCMin (n=4)
Wi (n=4)
Wi_APCMin (n=7)
WT (n=4)
condition

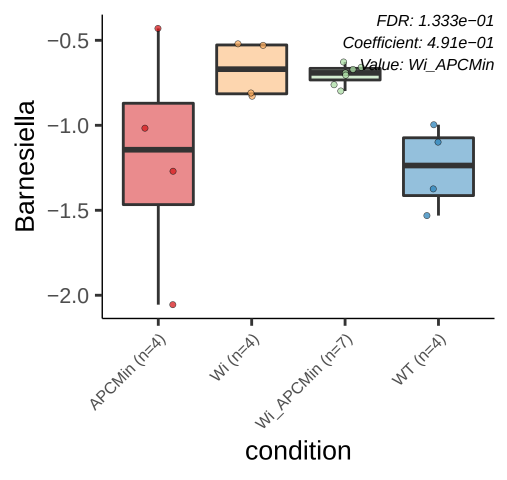

FDR: 1.333e−01
Coefficient: 4.91e−01
Value: Wi_APCMin
Barnesiella
−0.5
−1.0
−1.5
−2.0
APCMin (n=4)
Wi (n=4)
Wi_APCMin (n=7)
WT (n=4)
condition

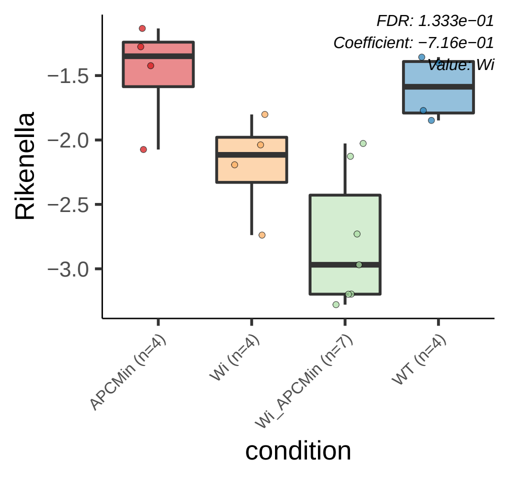
FDR: 1.333e−01
Coefficient: −7.16e−01
Value: Wi
Rikenella
−1.5
−2.0
−2.5
−3.0
APCMin (n=4)
Wi (n=4)
Wi_APCMin (n=7)
WT (n=4)
condition

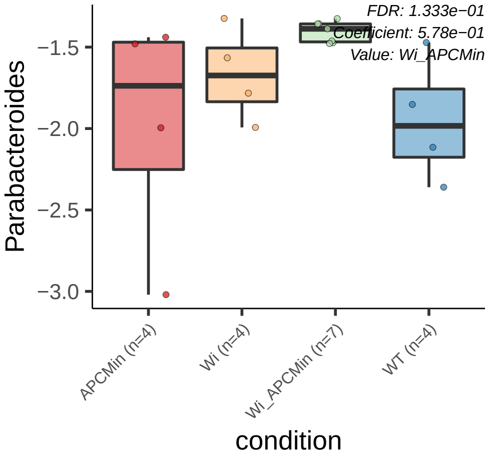

FDR: 1.333e−01
Coefficient: 5.78e−01
Value: Wi_APCMin
Parabacteroides
−1.5
−2.0
−2.5
−3.0
APCMin (n=4)
Wi (n=4)
Wi_APCMin (n=7)
WT (n=4)
condition

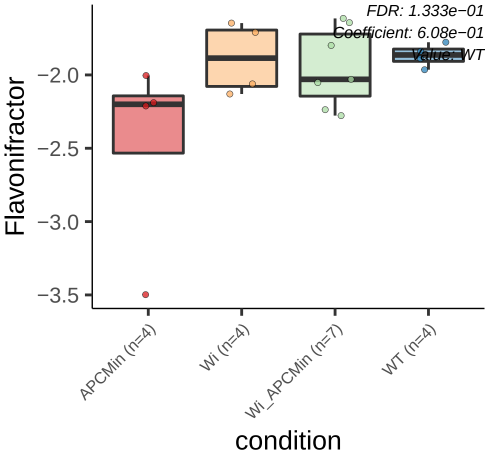

FDR: 1.333e−01
Coefficient: 6.08e−01
Value: WT
Flavonifractor
−2.0
−2.5
−3.0
−3.5
APCMin (n=4)
Wi (n=4)
Wi_APCMin (n=7)
WT (n=4)
condition

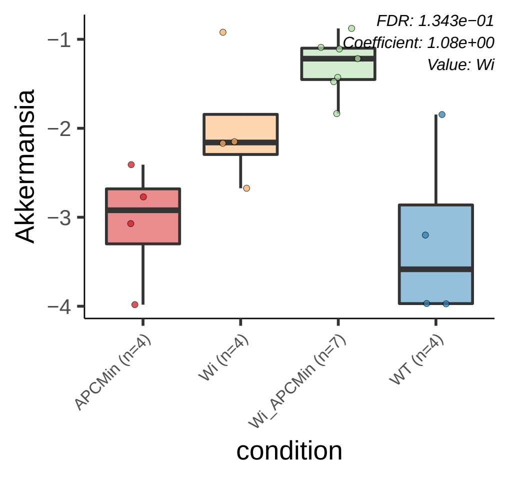
FDR: 1.343e−01
Coefficient: 1.08e+00
Value: Wi
Akkermansia
−1
−2
−3
−4
APCMin (n=4)
Wi (n=4)
Wi_APCMin (n=7)
WT (n=4)
condition

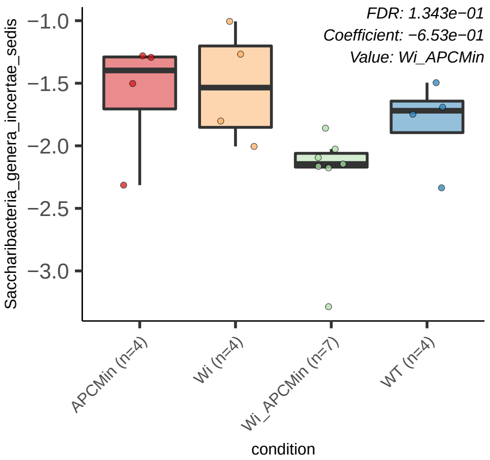

FDR: 1.343e−01
Coefficient: −6.53e−01
Value: Wi_APCMin
Saccharibacteria_genera_incertae_sedis
−1.0
−1.5
−2.0
−2.5
−3.0
APCMin (n=4)
Wi (n=4)
Wi_APCMin (n=7)
WT (n=4)
condition

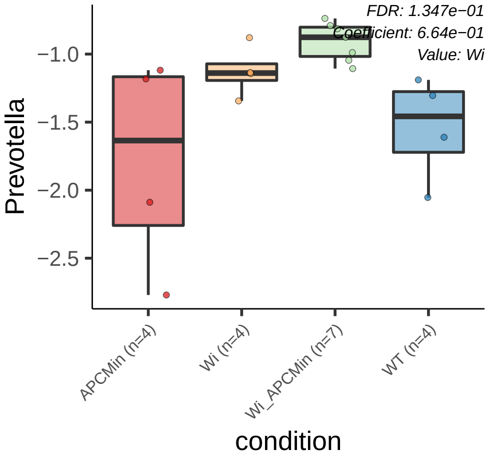
FDR: 1.347e−01
Coefficient: 6.64e−01
Value: Wi
Prevotella
−1.0
−1.5
−2.0
−2.5
APCMin (n=4)
Wi (n=4)
Wi_APCMin (n=7)
WT (n=4)
condition

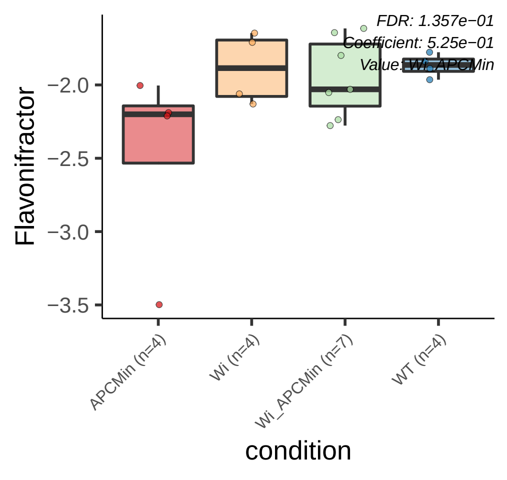

FDR: 1.357e−01
Coefficient: 5.25e−01
Value: Wi_APCMin
Flavonifractor
−2.0
−2.5
−3.0
−3.5
APCMin (n=4)
Wi (n=4)
Wi_APCMin (n=7)
WT (n=4)
condition

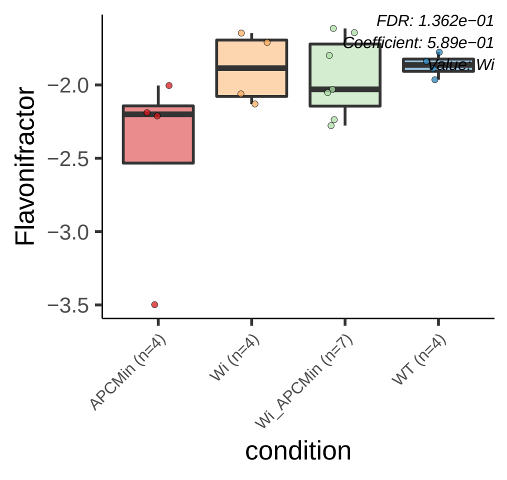
FDR: 1.362e−01
Coefficient: 5.89e−01
Value: Wi
Flavonifractor
−2.0
−2.5
−3.0
−3.5
APCMin (n=4)
Wi (n=4)
Wi_APCMin (n=7)
WT (n=4)
condition

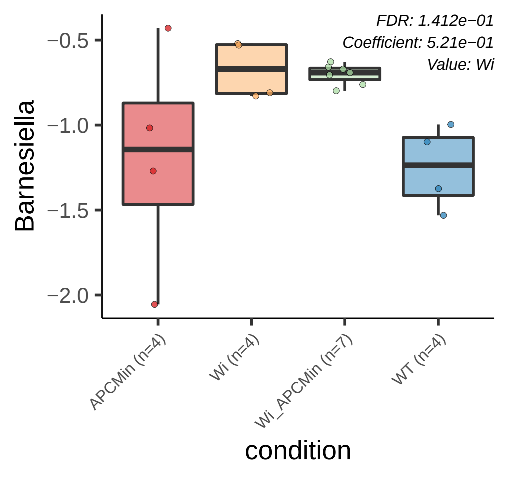
FDR: 1.412e−01
Coefficient: 5.21e−01
Value: Wi
Barnesiella
−0.5
−1.0
−1.5
−2.0
APCMin (n=4)
Wi (n=4)
Wi_APCMin (n=7)
WT (n=4)
condition

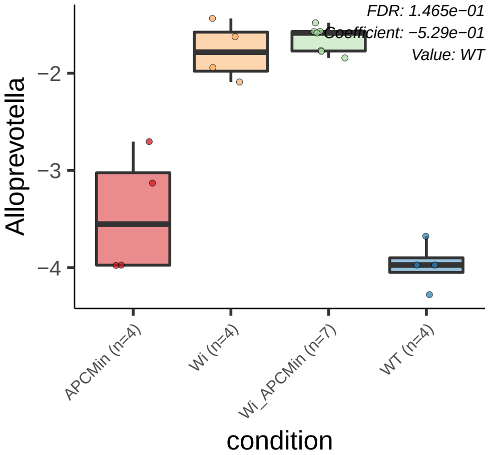

FDR: 1.465e−01
Coefficient: −5.29e−01
Value: WT
Alloprevotella
−2
−3
−4
APCMin (n=4)
Wi (n=4)
Wi_APCMin (n=7)
WT (n=4)
condition

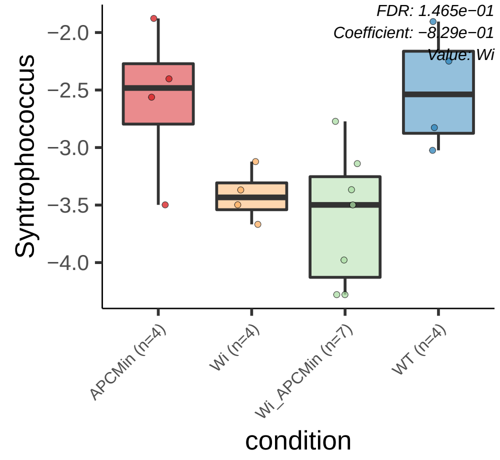

FDR: 1.465e−01
Coefficient: −8.29e−01
Value: Wi
Syntrophococcus
−2.0
−2.5
−3.0
−3.5
−4.0
APCMin (n=4)
Wi (n=4)
Wi_APCMin (n=7)
WT (n=4)
condition

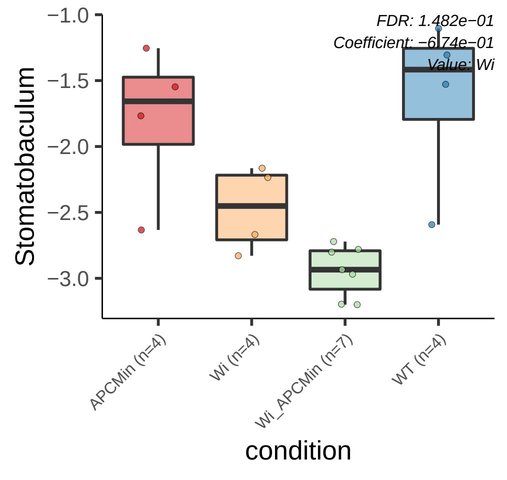

FDR: 1.482e−01
Coefficient: −6.74e−01
Value: Wi
Stomatobaculum
−1.0
−1.5
−2.0
−2.5
−3.0
APCMin (n=4)
Wi (n=4)
Wi_APCMin (n=7)
WT (n=4)
condition

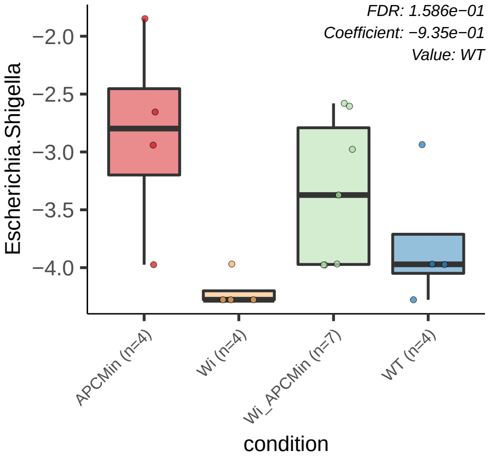

FDR: 1.586e−01
Coefficient: −9.35e−01
Value: WT
Escherichia.Shigella
−2.0
−2.5
−3.0
−3.5
−4.0
APCMin (n=4)
Wi (n=4)
Wi_APCMin (n=7)
WT (n=4)
condition

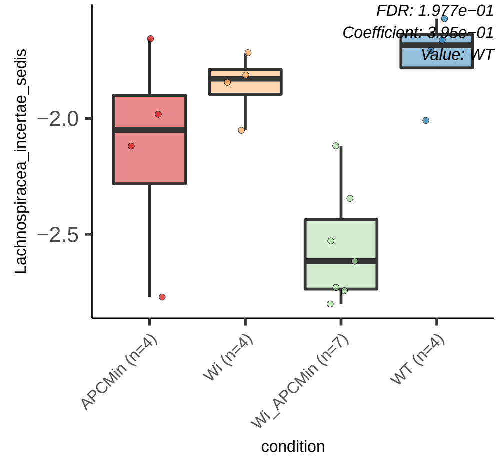

FDR: 1.977e−01
Coefficient: 3.95e−01
Value: WT
Lachnospiracea_incertae_sedis
−2.0
−2.5
APCMin (n=4)
Wi (n=4)
Wi_APCMin (n=7)
WT (n=4)
condition

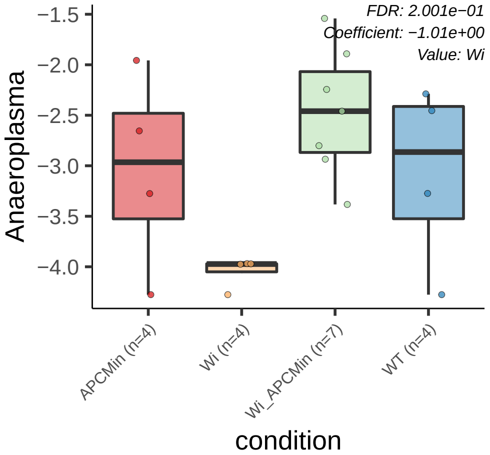

FDR: 2.001e−01
Coefficient: −1.01e+00
Value: Wi
Anaeroplasma
−1.5
−2.0
−2.5
−3.0
−3.5
−4.0
APCMin (n=4)
Wi (n=4)
Wi_APCMin (n=7)
WT (n=4)
condition

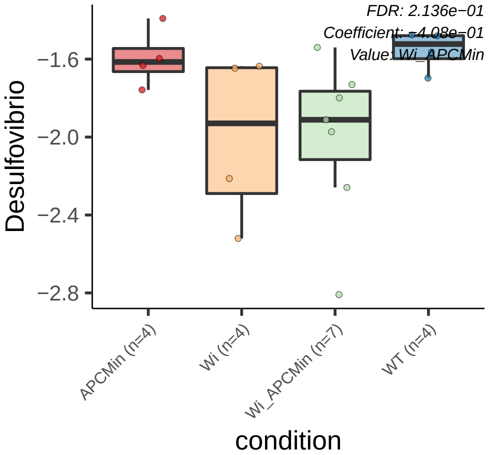
FDR: 2.136e−01
Coefficient: −4.08e−01
Value: Wi_APCMin
Desulfovibrio
−1.6
−2.0
−2.4
−2.8
APCMin (n=4)
Wi (n=4)
Wi_APCMin (n=7)
WT (n=4)
condition

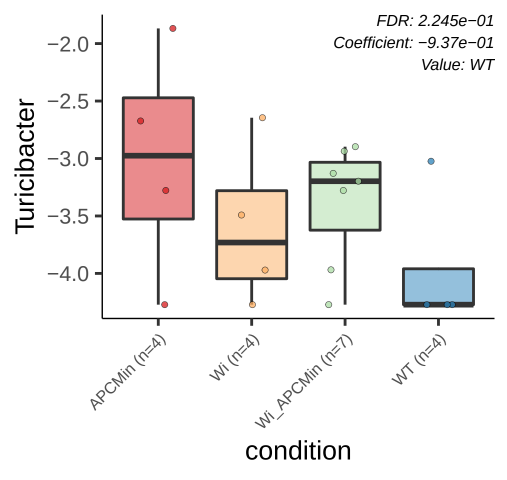
FDR: 2.245e−01
Coefficient: −9.37e−01
Value: WT
Turicibacter
−2.0
−2.5
−3.0
−3.5
−4.0
APCMin (n=4)
Wi (n=4)
Wi_APCMin (n=7)
WT (n=4)
condition

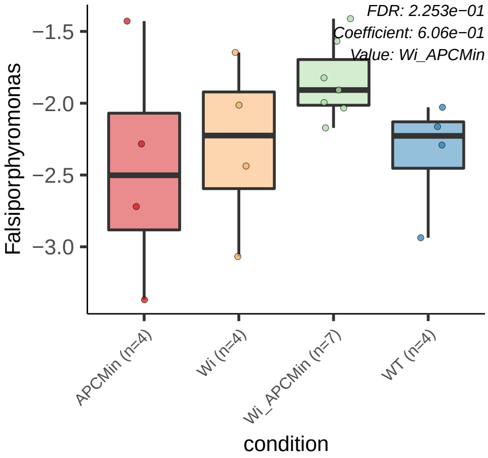
FDR: 2.253e−01
Coefficient: 6.06e−01
Value: Wi_APCMin
Falsiporphyromonas
−1.5
−2.0
−2.5
−3.0
APCMin (n=4)
Wi (n=4)
Wi_APCMin (n=7)
WT (n=4)
condition

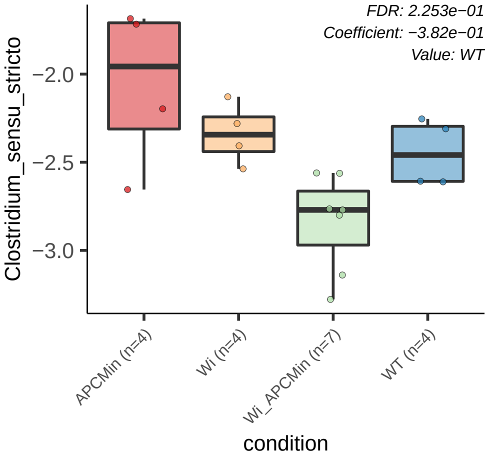
FDR: 2.253e−01
Coefficient: −3.82e−01
Value: WT
Clostridium_sensu_stricto
−2.0
−2.5
−3.0
APCMin (n=4)
Wi (n=4)
Wi_APCMin (n=7)
WT (n=4)
condition

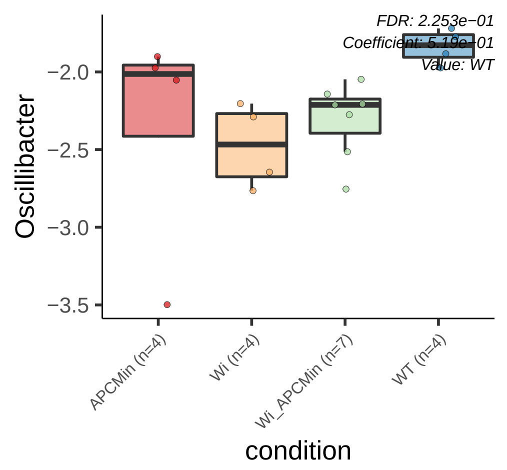

FDR: 2.253e−01
Coefficient: 5.19e−01
Value: WT
Oscillibacter
−2.0
−2.5
−3.0
−3.5
APCMin (n=4)
Wi (n=4)
Wi_APCMin (n=7)
WT (n=4)
condition

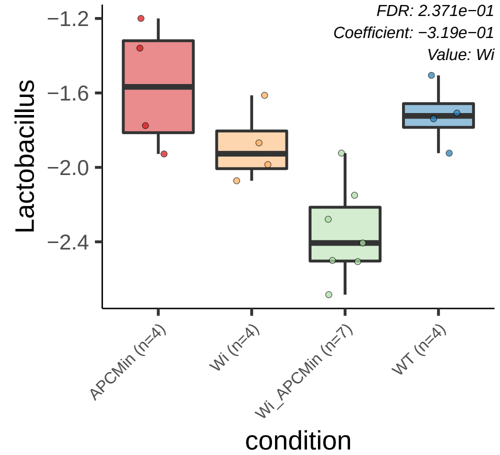

FDR: 2.371e−01
Coefficient: −3.19e−01
Value: Wi
Lactobacillus
−1.2
−1.6
−2.0
−2.4
APCMin (n=4)
Wi (n=4)
Wi_APCMin (n=7)
WT (n=4)
condition

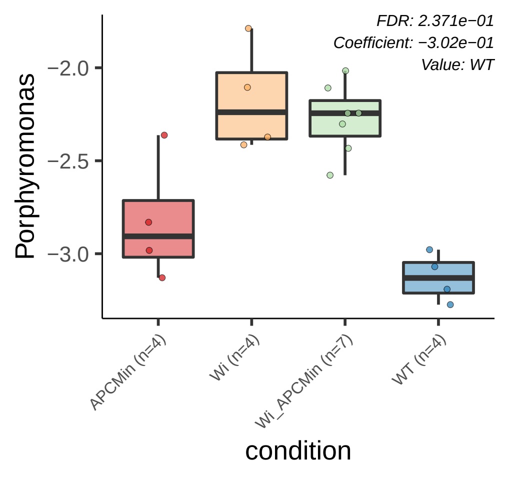

FDR: 2.371e−01
Coefficient: −3.02e−01
Value: WT
Porphyromonas
−2.0
−2.5
−3.0
APCMin (n=4)
Wi (n=4)
Wi_APCMin (n=7)
WT (n=4)
condition

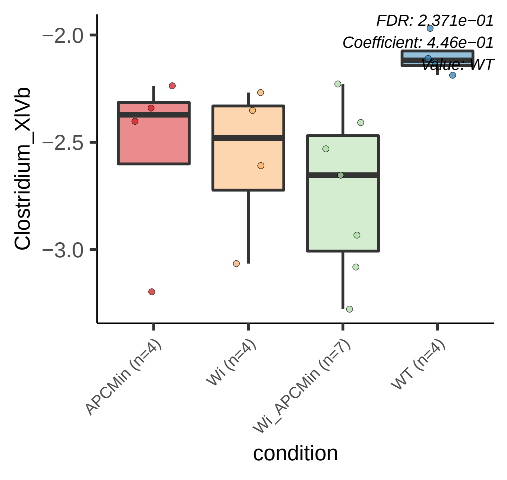
FDR: 2.371e−01
Coefficient: 4.46e−01
Value: WT
Clostridium_XlVb
−2.0
−2.5
−3.0
APCMin (n=4)
Wi (n=4)
Wi_APCMin (n=7)
WT (n=4)
condition

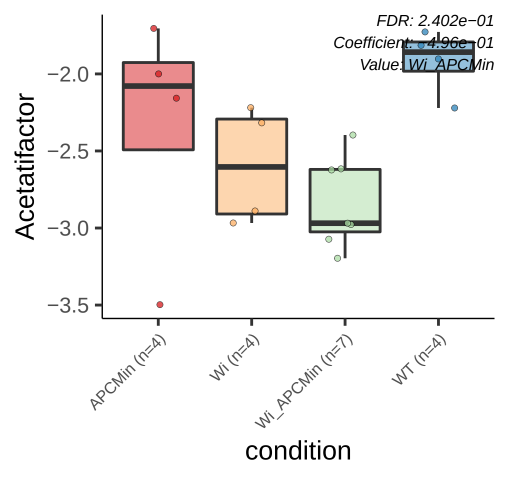

FDR: 2.402e−01
Coefficient: -4.96e-01
Value: Wi_APCMin
Acetatifactor
−2.0
−2.5
−3.0
−3.5
APCMin (n=4)
Wi (n=4)
Wi_APCMin (n=7)
WT (n=4)
condition

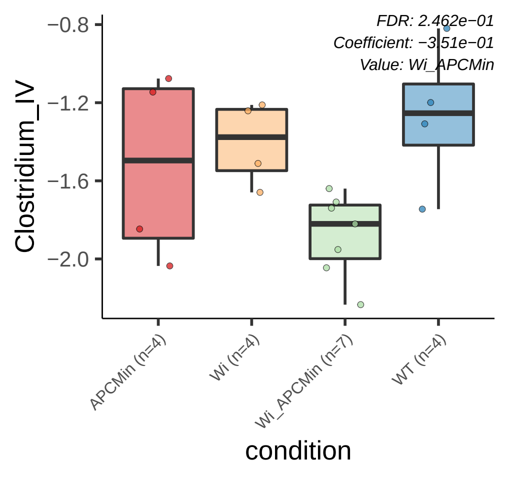

FDR: 2.462e−01
Coefficient: −3.51e−01
Value: Wi_APCMin
Clostridium_IV
−0.8
−1.2
−1.6
−2.0
APCMin (n=4)
Wi (n=4)
Wi_APCMin (n=7)
WT (n=4)
condition

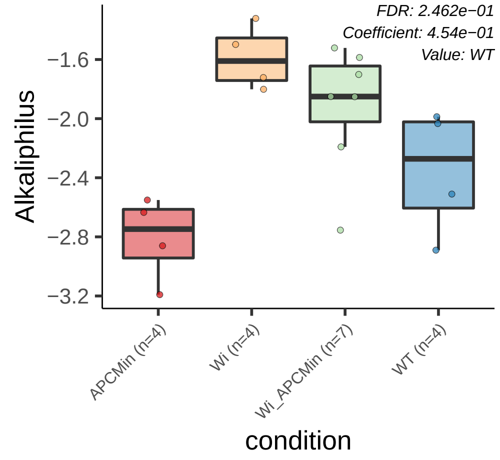

FDR: 2.462e−01
Coefficient: 4.54e−01
Value: WT
Alkaliphilus
−1.6
−2.0
−2.4
−2.8
−3.2
APCMin (n=4)
Wi (n=4)
Wi_APCMin (n=7)
WT (n=4)
condition
