# Supplementary material for: Dysbiosis Triggers ACF Development in Genetically Predisposed Subjects
Source: Cancers (Basel). 2021 Jan 14;13(2):283. doi: 10.3390/cancers13020283 (PMC7828790; doi:10.3390/cancers13020283)
Supplement: Supplementary file 1 [file cancers-13-00283-s001.zip › supplemental figures/Supplementary Figure Legends.docx]

**SUPPLEMENTARY FIGURE LEGENDS**

**Figure S1:** *Firmicutes* to *Bacteroidetes* ratios was calculated for 8-week-old Winnie-APC^Min/+^ (Wi_APCMin), Winnie (Wi), APC^Min/+^(APCMin), and C57BL/6J (WT). (*) p = 0.0181 (Kruskal-Wallis test corrected with Dunn’s multiple comparisons test).

**Figure S2:** Positive (coef+) or negative (coef-) associations of bacterial abundances (16rDNA gene amplicon) at family level and mouse genotypes: Winnie-APC^Min/+^(Wi_APCMin), Winnie (Wi), APC^Min/+^(APCMin), and C57BL/6J (WT).

**Figure S3:** Positive (coef+) or negative (coef-) associations of bacterial abundances (16rDNA gene amplicon) at genus level and mouse genotypes: Winnie-APC^Min/+^(Wi_APCMin), Winnie (Wi), APC^Min/+^(APCMin), and C57BL/6J (WT).
