# Supplementary figures and images for: Dysbiosis Triggers ACF Development in Genetically Predisposed Subjects
Source: Cancers (Basel). 2021 Jan 14;13(2):283. doi: 10.3390/cancers13020283 (PMC7828790; doi:10.3390/cancers13020283)

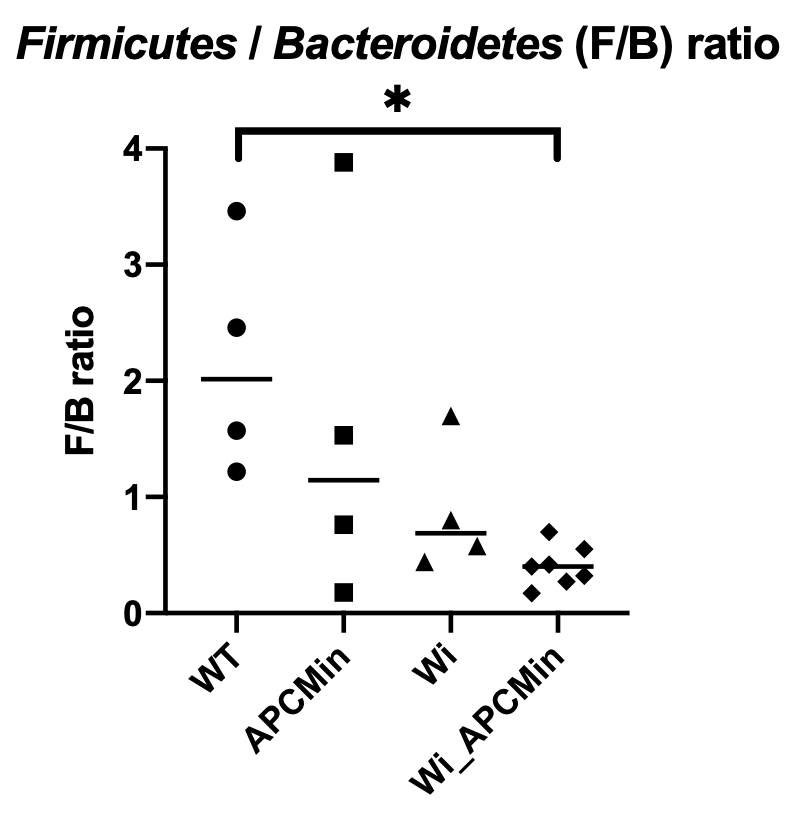

Supplement: Supplementary file 1 [file cancers-13-00283-s001.zip › supplemental figures/Figure S1.png]
